# Metal-Insulator Transition of strained SmNiO_3_ Thin Films: Structural, Electrical and Optical Properties

**DOI:** 10.1038/srep40915

**Published:** 2017-01-18

**Authors:** B. Torriss, J. Margot, M. Chaker

**Affiliations:** 1 INRS-EMT, 1650 Lionel-Boulet, C. P. 1020, Varennes Québec, J3X 1S2, Canada; 2 Département de Physique, Université de Montréal, CP. 6128 Succ. Centre-ville, Montréal, Québec H3C 3J7, Canada

## Abstract

Samarium nickelate (SmNiO_3_) thin films were successfully synthesized on LaAlO_3_ and SrTiO_3_ substrates using pulsed-laser deposition. The Mott metal-insulator (MI) transition of the thin films is sensitive to epitaxial strain and strain relaxation. Once the strain changes from compressive to tensile, the transition temperature of the SmNiO_3_ samples shifts to slightly higher values. The optical conductivity reveals the strong dependence of the Drude spectral weight on the strain relaxation. Actually, compressive strain broadens the bandwidth. In contrast, tensile strain causes the effective number of free carriers to reduce which is consistent with the d-band narrowing.

Perovsike nickelates RNiO_3_ (R = rare earth) provide a remarkable example of materials to achieve a good understanding of the interdependence between structural, electronic and magnetic properties by simply changing the R cation^1^,^2^. Except for R = La, the materials of the RNiO_3_ series exhibit steep metal-insulator (MI) transition and antiferromagnetic ordering^3^,^4^. The MI transition can be tuned by chemical doping^5^,^6^,^7^, applied hydrostatic pressures^8^,^9^,^10^ and electric field^11^, which opens new opportunities for future electronic devices^12^,^13^. According to the diagram proposed by Zaanen *et al*.^14^, RNiO_3_ lies in the charge-transfer region in which the band gap between the nickel 3d-band and the oxygen p-band is smaller than the on-site Coulomb interaction (U_dd_). The change of both Ni-O-Ni bond angle and NiO length bond with increasing temperature reduces the band gap, hence leading to a metallic phase. Some studies have shown that charge ordering plays a role in the MI transition. Indeed, a long-range charge imbalance characterized by the parameter δ, i.e. 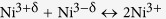 has been observed in the insulating regime, while nickel is uniformly trivalent in the metallic state^15^,^16^,^17^. On the other hand, the elements of RNiO_3_ have been classified as small or negative charge transfer systems^18^,^19^,^20^. In this case, some holes are transferred from the 3d orbitals to the 2p orbitals and the ground state can thus be described as a mixture of d^7^ and d^8^L configurations instead of the purely ionic d^7^ configuration. Recent dynamical mean field theory (DMFT) calculations mention the importance of ligand holes in the electronic structure. For example, Park *et al*.^19^ explained the insulating phase as a “site-selective” Mott phase associated with long bound sites while Johnston *et al*.^21^ suggest a novel charge ordering 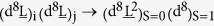, where S is the total spin. According to this picture, the long-bond octahedra are associated with the d^8^ configuration (with a large local moment) and the short-bond ones with 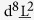 (with the Ni local moment screened by the two ligand holes). Furthermore, the charge ordering is achieved without any significant movement of charge^21^.

The crystallization of bulk RNiO_3_ in the perovskite structure is normally achieved at high temperatures and oxygen pressures. However, another way to stabilize the nickelate phase is to use the template effect of a perovskite substrate with minimal lattice mismatch. Several research groups are interested to understand the relationship between the nature of epitaxial strain and the MI transition due to changes in the nickel oxidation state. For example, in-plane compressive strain maintains the oxidation state in the SNO films, resulting in a steep MI transition^22^,^23^. On the other hand, the tensile strain induces the creation of oxygen vacancies, which leads to the annihilation of the MI transition. This has been observed for example in the case of epitaxial SNO grown on SrTiO_3_ (STO) substrate^24^,^25^. However, our team has recently demonstrated the capability to grow epitaxial SNO thin films under tensile strain on STO substrate with a steep MI transition at about 394 K^26^.

In this work, we examine how the strain relaxation (compressive/tensile) affects the electronic properties of epitaxial SNO thin films grown on LaAlO_3_ (LAO, compressive) and STO (tensile) as well as their optical characteristics in the UV-visible and infrared spectrum.

## Results

**Figure 1:**
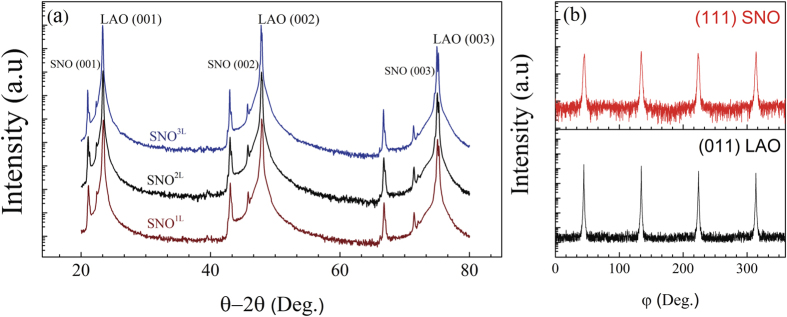
(**a**) XRD spectra of SNO thin films on LAO with different film thicknesses. (**b**) φ scans performed on (011) pseudocubic reflection of LAO and (111) orthorhombic reflection of SNO.

**Figure 2:**
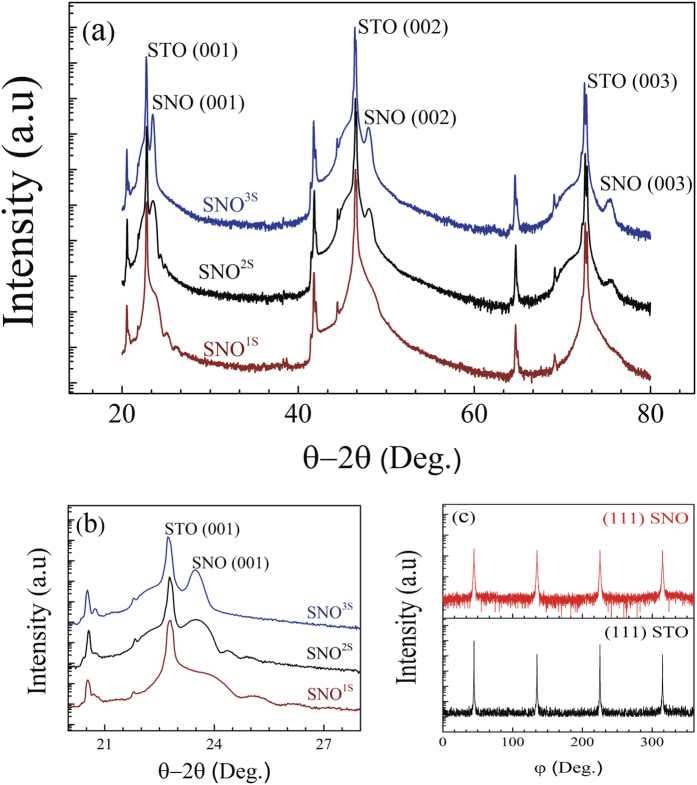
(**a**) X-ray spectra of SNO films grown on STO with different film thicknesses. (**b**) details of the X-ray spectra around the (001) peak of SNO films grown on STO. (**c**) φ scans performed on (111)_STO_ and (111)_SNOpc_ reflections.

At room temperature, bulk SNO possesses an orthorhombic (orth) structure and lattice parameters a_0_ = 5.328 Å, b_0_ = 5.437 Å and c_0_ = 7.567 Å. The SNO unit cell can be described by a pseudo-cubic unit with a_pc_ = 3.799 Å, yielding a volume of 54.82 Å^3^ 27. The LAO (001) and STO substrates (001) provide −0.26% (compressive strain) and +2.7% (tensile strain) lattice mismatch, respectively. Figure 1a displays the 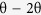 x-ray pattern of SNO thin films of thickness equal to 8.5 (SNO^1L^), 16 (SNO^2L^) and 63 nm (SNO^3L^) grown on LAO substrates (see definitions of SNO exponents in the Methods section). We only observe the (00 L) reflection of LAO due to its very close lattice match with SNO. For the samples grown on STO (Fig. 2a), the sole presence of (00 L) peaks of SNO and STO in the x-ray diffraction scans indicates a textured growth. In Fig. 2, Laue fringes are clearly visible for the SNO^1S^ and SNO^2S^ samples. Their presence indicates a uniform thickness with two well–defined interfaces (SNO/STO and SNO/Air). In order to investigate the epitaxial relation between SNO and STO, XRD φ measurements were performed on the (111)_SNOorth_/(011)_LAOpc_ and (111)_STO_/(111)_SNOpc_ reflections (Figs 1b and 2c). The SNO peaks are superimposed with those from the LAO (Fig. 1b) and STO (Fig. 2c) substrates confirming the cube-on-cube growth of our films. In addition, the atomic force microscopy (AFM) images of 8.5 nm-thick SNO films grown on LAO and STO (data not presented here) show atomically smooth surfaces with a root mean square (RMS) roughness of ~0.2 *nm* and ~0.3 *nm*, respectively.

**Figure 3:**
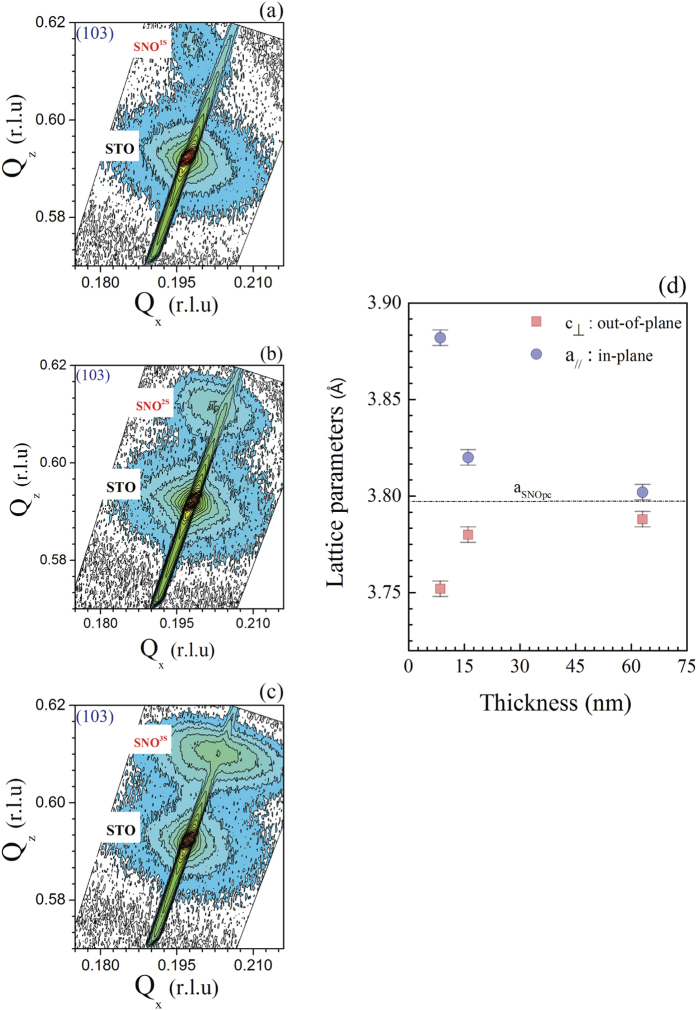
(**a**–**c**) Reciprocal lattice maps around (103) Bragg reflections of SNO thin films on STO with different film thicknesses. (**d**) Lattice parameters for SNO films on STO as a function of thickness.

To analyse the strain behaviour of the films grown on STO, we measured the reciprocal space maps (RSM) asymmetrical Bragg reflection (103) as illustrated in Fig. 3a to Fig. 3c. Figure 3d shows the dependence of the lattice parameters on the film thickness. From this figure, it can be concluded that the film thickness significantly influences the strain state of the SNO films deposited on STO. For the SNO^1S^ sample, the in-plane lattice parameter is larger than the bulk value while the out-of-plane lattice parameter is lower. As the film thickness increases, the SNO film strain reduces so that both in-plane and out-of-plane lattice parameters tend towards their bulk values.

**Figure 4:**
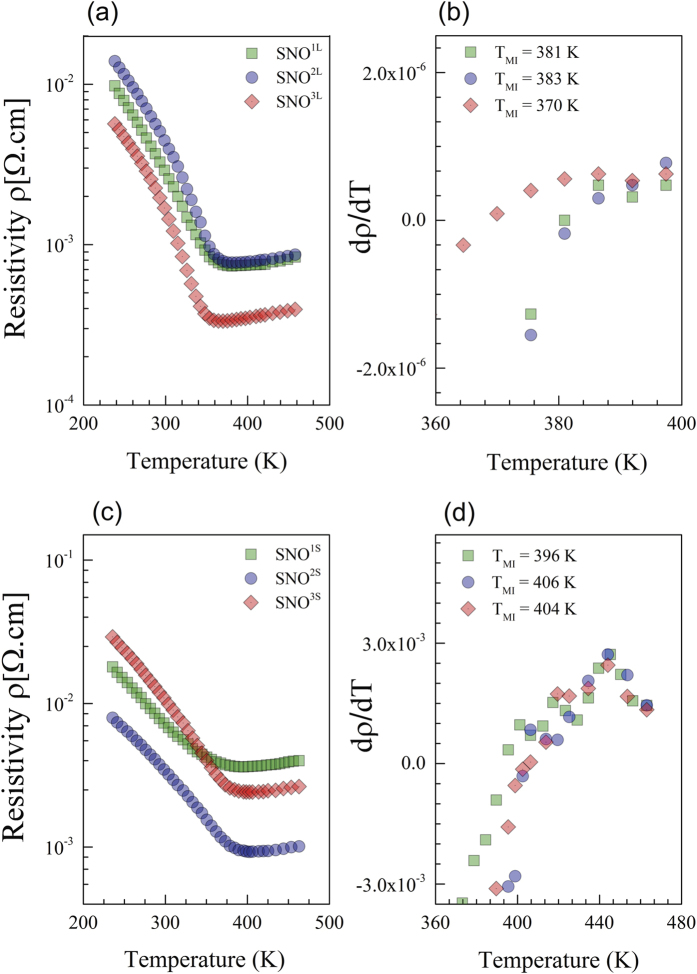
Temperature dependence of the resistivity for SNO thin films on LAO (**a**) and STO (**c**) substrates. Derivative of resistivity 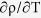 of SNO films on LAO (**b**) and STO (**d**). T_MI_ corresponds to the temperature at which the sign of 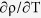 changes and its uncertainty is defined by the temperature step size, 5 K.

The temperature dependence of the resistivity of SNO films grown on LAO and STO is shown in Fig. 4a and c, respectively. All the samples exhibit a MI transition. The temperature corresponding to the MI transition is attained when the sign of the resistivity derivative changes (see Fig. 4b and d). For SNO films under compressive strain (i.e. grown on LAO), values of T_MI_ equal to 381 K, 383 K and 370 K are obtained for SNO^1L^, SNO^2L^ and SNO^3L^, respectively. These transition temperatures are consistent with those reported in previous studies^28^,^29^,^30^. Recently, theoretical calculations have revealed that compressive strain is reduced by straightening the Ni-O-Ni angle, which increases the orbital overlap and modifies the bandwidth. Such bond straightening leads to a continuous shift of the MIT from T_MI_ = 400 K to T_MI_ = 0 K^30^.

For the SNO films under tensile strain grown on STO, the MI transition is found to be about 396 K, 406 K and 404 K for SNO^1S^, SNO^2S^ and SNO^3S^, respectively. Furthermore, we observe that the MI transition is broader while the metallic state is significantly resistive, particularly for the sample SNO^1S^. It is well known the oxygen non-stoichiometry results in an expansion of the cell volume and is accompanied by the suppression of MI transition^25^,^31^. Compared to the unit cell of SNO bulk (volume of 54.8 Å^3^), the thinner sample SNO^1S^ exhibits a larger volume (56.54 Å^3^), which suggests that the creation of oxygen vacancies is the mechanism leading to tensile strain and that the oxygen vacancies are mainly close to the interface SNO/STO^32^. When the sample thickness is increased, the unit cell volume of SNO^2S^ and SNO^3S^ shrinks to 55.1 and 54.75 Å^3^, respectively. These values are close to that of the stoichiometric bulk sample, which suggests that the amount of oxygen vacancies in the films has become negligible. This observation indicates that mechanical rather than chemical relaxation (oxygen vacancies) is the dominant mechanism of strain relaxation. Therefore, it can be concluded that the tensile strain generated at the interface reduces the Ni-O-Ni bond angle unlike the case of compressive strain and increases the buckling of the NiO_6_ octahedra, which results in less orbital overlap, i.e. in a narrowing of the d-band^33^,^34^.

**Figure 5:**
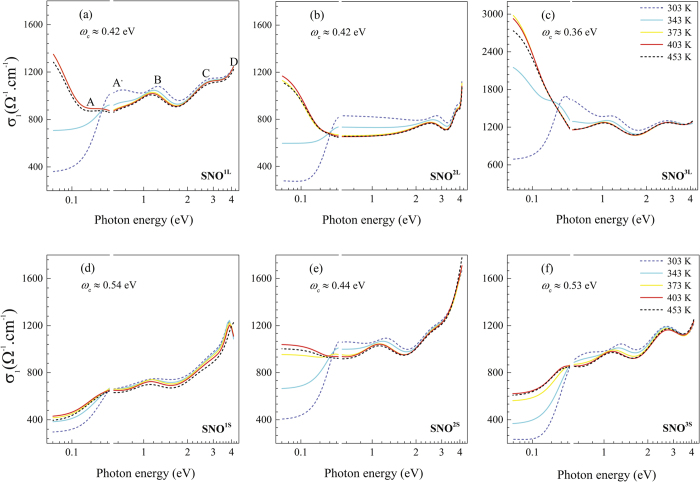
Optical conductivity as a function of photon energy for SNO thin films on LAO (**a**–**c**) and on STO (**d**–**f**) substrates at different temperatures.

In Fig. 5, the real part of the optical conductivity σ_1_(ω) of the all samples is displayed as a function of the photon energy for different values of the temperature. The optical conductivity is related to the complex dielectric function though the equation 
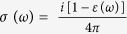
. For all the samples, a Drude contribution and four peaks (A, B, C, and D) corresponding to interband transitions can be identified. According to the band structure calculations for SNO crystal^35^, we suggest that the A and B features are due to the transitions between the occupied and unoccupied e^*^_g_ orbitals. The excitations from the t^*^_g_ and the nonbonding O_2p_ to the e^*^_g_ orbitals are denominated C. The optical conductivity of the various samples spectra is remarkably different at high energy. For example, at 4 eV, the D peak is evident only for the thinner film SNO^1S^ grown on STO. It can be attributed to a transition between t_2g_ and e^*^_g_ levels.

In the case of SNO grown on LAO, the Drude-like peak present at very low energy disappears rapidly when the temperature is decreased towards 303 K while a new peak (denoted as A’ in Fig. 5) emerges at 0.6 eV. The transfer of spectral weight (SW) from the Drude resonance to finite energy peak is characteristic of charge-ordered systems with a weak electron-lattice coupling^36^,^37^. Indeed, Stewart *et al*. have concluded that both charge ordering and Mott physics occur in the insulating state for NdNiO_3_ thin film on STO substrate^38^. Furthermore, the recent DMFT calculations of bulk SNO at low temperature enable us to identify the dominant features of the optical conductivity. Indeed, Ruppen *et al*.^39^ demonstrated the peak B (1.4 eV) is due to the optical transition across the Peierls pseudogap, while the peak A’ corresponds to the transition across the insulating gap. The presence of these peaks results from the combined effect of bond disproportionation and Mott physics associated with half of the disproportionated sites^39^.

To determine the changes driven by the temperature in the electronic structure across the MI transition of the SNO films, we have estimated the effective number of free carriers (N_eff_) defined as:


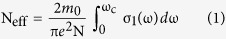


**Figure 6:**
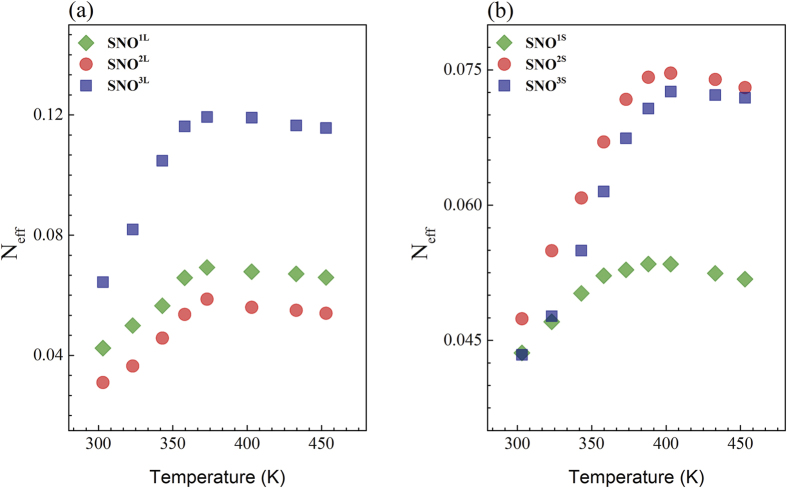
Temperature dependence of the effective number of electrons per atom of Ni for SNO films on LAO (**a**) and on STO (**b**).

Here, *m*_0_ is the free electron mass and N the number of Ni atoms per unit volume, while ω_c_ is the cut-off frequency. The values of ω_c_ are indicated in Fig. 5 for each sample. Figures 6a and b show the value of N_eff_ as a function of temperature for the samples SNO on LAO and STO, respectively. As it can be seen, in both cases, N_eff_ reaches a saturation value close to 388 K. Below 388 K, N_eff_ decreases with decreasing temperature due to the gap formation^40^. The number of electrons localized at the MI transition can be calculated as 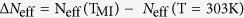. For the films deposited on LAO, we obtain values of ΔN_eff_ = 0.026, 0.027 and 0.054 for SNO^1L^, SNO^2L^ and SNO^3L^, respectively. For those deposited on STO, we obtain 0.009, 0.027 and 0.028 for SNO^1S^, SNO^2S^ and SNO^3S^ respectively. The conduction properties in the metallic state are correlated to ΔN_eff_, which represents the spectral weight 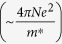. Assuming one conduction electron per Ni site, this value yields an effective mass 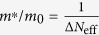 where m^*^ is the effective mass of conduction carriers. A small value of ΔN_eff_ corresponds a larger 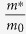, which characterizes a narrow conduction band composed of Ni 3d and O_2p_ levels, and to larger mass enhancement by strong electron-correlation effects. The mass enhancement is higher in the SNO films on STO (tensile strain) comparatively to the films grown on LAO (compressive strain).

## Discussion

The strain relaxation of the SNO films changes the lattice constants, which results in modifications of the Ni-O and Sm-O bonds lengths and/or of the Ni-O-Ni band angle. Such variation of the SNO unit cell leads to modifications of the electron bandwidth and influences the electrical transport and infrared optical properties. In-plane compressive strain maintains the oxidation state in the SNO films, resulting in a steep MI transition. The decrease of T_MI_ when the film thickness increases indicates that the SNO lattice is still able to sustain the epitaxial constraint by straightening the Ni-O-Ni angle, which broadens the bandwidth of the system.

As for the tensile relaxation characterizing SNO films deposited on STO, it is accompanied by the formation of oxygen vacancies at the interface. In our case, all the corresponding films exhibit a MI transition close to 400 K but smeared as compared to SNO deposited on LAO. Let us emphasize that in the literature, the role of oxygen vacancies in the perovskites nickelates is unclear whether the vacancies increase T_MI_^41^ or leave it unaffected^31^. The enhanced tensile strain associated with increasing film thickness is consistent with a narrowing of the band and an increase of the electron correlations. This tensile strain is accommodated by buckling of the NiO_6_ octahedra, which results in reduced orbital overlap and in the Drude spectral weight in the metallic state.

## Methods

In this study, SNO thin films were grown on *c*- axis oriented LAO and STO single crystal substrates by pulsed laser ablation of a stoichiometric target (99.99% Kurt J. Lesker). To ensure atomically flat terraces, STO substrates were etched for 30 s in buffered HF solution (NH_4_F: HF = 6:1), rinsed with distilled water and dried under a nitrogen stream. Finally, the substrates were heated at 1050 °C for 30 min in an oxygen flow. In the case of LAO, the substrates were annealed at 1100 °C for 12 h in a flowing O_2_ atmosphere. The samples were prepared in a high vacuum deposition system filled with an oxygen pressure of 300 mTorr. The laser fluence on the target was 2 J/cm^2^ while the substrate temperature was kept at 600 °C during the deposition. The SNO films were *in situ* annealed during 30 min and cooled at 5 °C/min at the same oxygen pressure. We characterized six samples denoted by SNO^1x^, SNO^2x^ and SNO^3x^, where 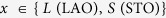, of thickness equal to 8.5 ± 0.4, 16 ± 0.2 and 63 ± 0.2 nm, respectively. Cross-sectional electron microscopy and x-ray reflectivity were used to determine the film thickness. The films structure and the epitaxial relationship with substrate were examined by four-circles x-ray diffraction (XRD). The resistivity of the thin films was measured as a function of the temperature from 235 to 463 K using the four-probe method. The resistivity *ρ* is deduced from the resistance by.


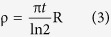


where t is the film thickness.

The optical characterization of the bare substrates and of the films was performed with two commercial Woollam ellipsometers from 40 meV to 4.13 eV. The Vase model based on a grating monochromator, covers the energy range between 0.62 and 4.13 eV, while the IR-Vase model was used for the range 0.04–0.61 eV. The measurements were carried out in the reflectivity configuration at an angle of incidence of 70° Spectroscopy ellipsometry converts the polarization state of the reflected light in two parameters ψ and Δ, which are related to the optical and structure properties of the samples, and defined by


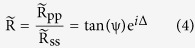


**Figure 7:**
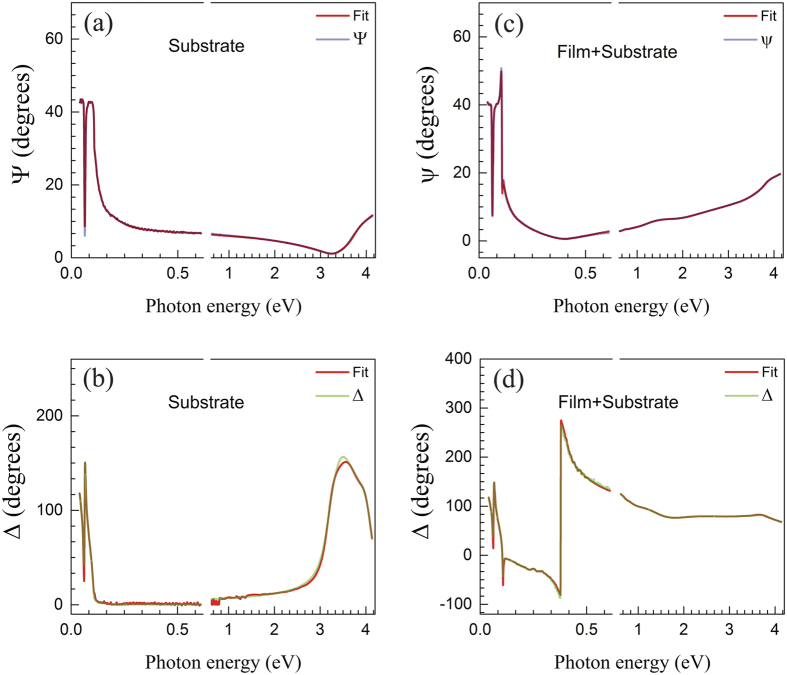
Experimental and best-fit calculated ellipsometry spectra of the STO substrate for ψ (**a**) and Δ (**b**) and of SNO films on STO for ψ (**c**) and Δ (**d**).

Where 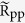 and 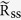 are the Fresnel reflection coefficients for p- and s- polarized light respectively. The experimental and best-fit calculated ellipsometry spectra for both ψ and Δ are displayed in Fig. 7a and b for the STO substrate, respectively. The corresponding results for SNO films on STO are shown in Fig. 7c and d.

The complex optical constants (subsequently the complex conductivities) of the SNO films were obtained by fitting numerically the data with a multilayers model involving the STO substrate, the SNO film and the transition layer. As LAO and STO have several far infrared phonons with strong temperature dependence, the samples and the substrates were measured and modeled at the same temperature. This allowed us to deduce accurate values of the complex conductivity of the SNO films from the fit of experimental data.

## Additional Information

**How to cite this article:** Torriss, B. *et al*. Metal-Insulator Transition of strained SmNiO_3_ Thin Films: Structural, Electrical and Infrared Optical Properties. *Sci. Rep.* **7**, 40915; doi: 10.1038/srep40915 (2017).

**Publisher's note:** Springer Nature remains neutral with regard to jurisdictional claims in published maps and institutional affiliations.
